# Effects of High Concentrations of Flumequine on *CYP* Gene Expression and Histopathology in Olive Flounder, *Paralichthys olivaceus*

**DOI:** 10.3390/ani15213125

**Published:** 2025-10-28

**Authors:** Gi Baeg Lee, Hyeon Ju Na, Ji-Min Jeong, Mun-Gyeong Kwon, Seong Don Hwang, Jung Soo Seo

**Affiliations:** 1 Department of Convergence Interdisciplinary Education of Maritime and Ocean Contents, Korea Maritime & Ocean University, Busan 49112, Republic of Korea; 2 Ocean Science and Technology School, Korea Maritime & Ocean University, Busan 49112, Republic of Korea; 3 Aquatic Disease Control Division, Fishery Products Quality Management Service, Busan 49111, Republic of Korea; 4 Department Division of Convergence on Marine Science, Korea Maritime & Ocean University, Busan 49112, Republic of Korea

**Keywords:** olive flounder, flumequine, cytochrome P450, histopathology

## Abstract

Flumequine is an antibiotic that is used to treat bacterial diseases in aquaculture. Fish express drug-metabolizing genes in response to antibiotic exposure. However, studies on the effects of high flumequine concentrations on drug metabolism genes and histopathology of the olive flounder are limited. To investigate the response of olive flounder to flumequine, we administered it at different concentrations. We analyzed the expression of drug metabolism genes (*CYP*) in the liver and histopathological lesions in the liver, spleen, and kidneys. The gene expression levels of *CYP* were higher at the highest flumequine concentration tested (4×) than at the lowest flumequine concentration (1×). The highest *CYP* gene expression level was observed for *CYP*2B4 (46.6-fold) at 4× flumequine compared to that in the control group. Hepatic atrophy, lymphocytic infiltration, and hematopoiesis were observed in the liver, spleen, and kidney at 4× flumequine between 3 and 24 h compared to 1× flumequine, respectively. These results contribute to a better understanding of drug metabolism and the general toxicity of pharmaceutical exposure in olive flounder.

## Simple Summary

Olive flounder is a valuable aquaculture species in South Korea, and a variety of antibiotics are used to treat bacterial diseases in aquatic organisms. In our study, we examined the effect of the quinolone-class flumequine on olive flounder. The results of the drug metabolism genes and histopathological symptoms indicate increased gene expression levels and a severe lesions tendency at the high concentration of flumequine (4×) compared to the low concentration (1×). This study may contribute to understanding the effects of flumequine on drug metabolism and general toxicity.

## 1. Introduction

Following the discovery of penicillin by Fleming, a variety of antibiotics have been developed and are used in aquaculture to treat bacterial diseases [1]. Since nalidixic acid was discovered in 1962, numerous synthetic derivatives of quinolone antibiotics have been developed to improve their antimicrobial efficacy [2]. A quinolone antibiotic, flumequine, is used to treat bacterial diseases. Flumequine inhibits bacterial activity by targeting enzymes involved in DNA replication, particularly DNA gyrase, thereby exerting its antibacterial effect by blocking bacterial DNA replication [3,4,5].

In South Korea, flumequine is used in aquaculture and veterinary medicine to treat bacterial diseases in aquatic organisms. Fish express drug-metabolizing genes in response to antibiotic exposure [6]. Drug metabolism refers to the chemical modification of drugs in the body. In a previous study of drug metabolism in fish, 94 cytochrome P450 (*CYP*) genes were identified in zebrafish (*Danio rerio*), a species commonly used in drug metabolism research [7]. *CYP* is a representative group of drug-metabolizing genes that play a critical role in the biotransformation and elimination of xenobiotics from the body. These genes are primarily expressed in the liver, which is one of the largest internal organs in fish and serves as a key site for the metabolism of absorbed antibiotics [8,9]. Furthermore, absorbed antibiotics have been shown to cause adverse effects on fish internal organs, including hepatocellular degeneration and necrosis [10,11,12]. The olive flounder, *Paralichthys olivaceus*, a benthic fish species belonging to the order Pleuronectiformes and family Paralichthyidae, is distributed along the entire coastline of Korea. The aquaculture industry experienced rapid growth in the 1980s, following the development of aquaculture production technologies and artificial seed production techniques [13]. Olive flounder is a commercially valuable aquaculture species that constituted 46% of the total domestic aquaculture production of fish in South Korea in 2022. In South Korea, flumequine antibiotics are restricted to oral administration, and the target species include olive flounder. Flumequine is one of the most frequently used antibiotics in aquaculture [4]. However, studies on *CYP* gene expression and histopathology of the olive flounder in response to high flumequine concentrations are limited. To investigate the response to flumequine in olive flounder, we examined drug metabolism-related genes and histopathological lesions following the administration of different concentrations of flumequine.

## 2. Materials and Methods

### 2.1. Experimental Animals and Drug

Healthy adult olive flounder weighing 408.2 ± 35.7 g (body length, 33.1 ± 1.5 cm) were purchased from a private farm in Pohang city, Gyeongbuk, Korea, and acclimated for two weeks under laboratory conditions. The absence of flumequine in the serum, muscle, and skin was confirmed by liquid chromatography coupled with tandem mass spectrometry (LC-MS/MS) method. Commercial flumequine was purchased from Komipharm (Siheung City, Gyeonggi-do, Republic of Korea). The recommended oral usage and dosage of flumequine is 120–200 g per body weight for 3–7 d, which has a raw material ingredient with 12–20 g of raw material ingredient per ton. To investigate the effect of flumequine, 20 fish in each of the six experimental groups (3, 6, 12, 24, 96, and 168 h) were orally administered a commercial pellet diet (CJ Feed Inc., Gunsan, Jeonbuk, Republic of Korea) containing flumequine at 1× and 4× doses for seven days. Diets were prepared by mixing flumequine with commercial pellets. Individual fish received flumequine at doses of 0.8 mg/g of fish body weight/day. Fish that were not treated with flumequine were used as the control group. All experimental feeds were supplied twice, and a total of 280 fish were used for the experiment. The water temperature in running water type culture tanks (1.5 ton) was maintained at 23 ± 0.5 °C using heat control systems (Aquatron System, Yoowon Electronics, Seoul, Republic of Korea). Experimental fish were humanely euthanized using MS-222 (ethyl 3-aminobenzoate methanesulfonate salt, Sigma-Aldrich, St. Louis, MO, USA). All the fish experiments were approved by the Institutional Animal Care and Use Committee (IACUC, NIFS-2019-6).

### 2.2. Expression Analysis of Drug Metabolism-Related Genes by Real-Time PCR

Total RNA was extracted from the liver using an RNeasy Mini Kit (Qiagen, Hilden, Germany) according to the manufacturer’s instructions. Total RNA was reverse transcribed using a Transcriptor First-Strand cDNA Synthesis Kit (Roche, Dublin, Ireland). Real-Time quantitative reverse transcription PCR (RT-qPCR) was used to evaluate gene expression levels using a 7500 Real-Time PCR System (Applied Biosystems, Waltham, MA, USA). RT-qPCR was performed with SYBR™ Green PCR Master Mix (Applied Biosystems, Waltham, MA, USA) using specific primer sets of the different cytochrome P-450 genes (Table 1) according to the manufacturer’s protocol. Expression of the target genes was normalized to an endogenous reference β-actin and presented as the subtraction of target CT values from β-actin CT values (ΔCT value). Comparison of gene expression between groups and calibrator was derived from subtraction of the calibrator ΔCT values from the target ΔCT values to give a ΔΔCT value. Relative gene expression was calculated to determine fold difference (2^−ΔΔCT^). The control group was used to perform relative quantification by comparison with the values of the 1× and 4× experimental groups at each time point (3, 6, 12, 24, 96, and 168 h). Significant differences between the flumequine-treated and control groups were determined using the SPSS Student’s *t*-test (Version 25.0, SPSS Inc., Chicago, IL, USA). All experiments were performed in triplicates.

### 2.3. Histopathological Analysis

For histopathological analysis, olive flounders from the control and experimental groups (3, 6, 12, 24, 96, and 168 h) were collected after administration of flumequine at different concentrations (1× and 4×). The fish were anesthetized using MS-222 (ethyl 3-aminobenzoate methanesulfonate salt) and the liver, spleen, and kidney were separated and fixed in 10% neutral buffered formalin. Tissue samples were dehydrated through an ethanol, cleared, and embedded in paraffin wax using a tissue processor (Leica TP1020, Leica Biosystems, Wetzlar, Germany). Tissue blocks were sectioned (4 µm thickness) using a microtome machine (Leica RM2235, Leica Biosystems, Wetzlar, Germany) and stained with hematoxylin and eosin (H&E) for microscopy. All experiments were performed in triplicates.

## 3. Results

### 3.1. Gene Expression Analysis

**Figure 1:**
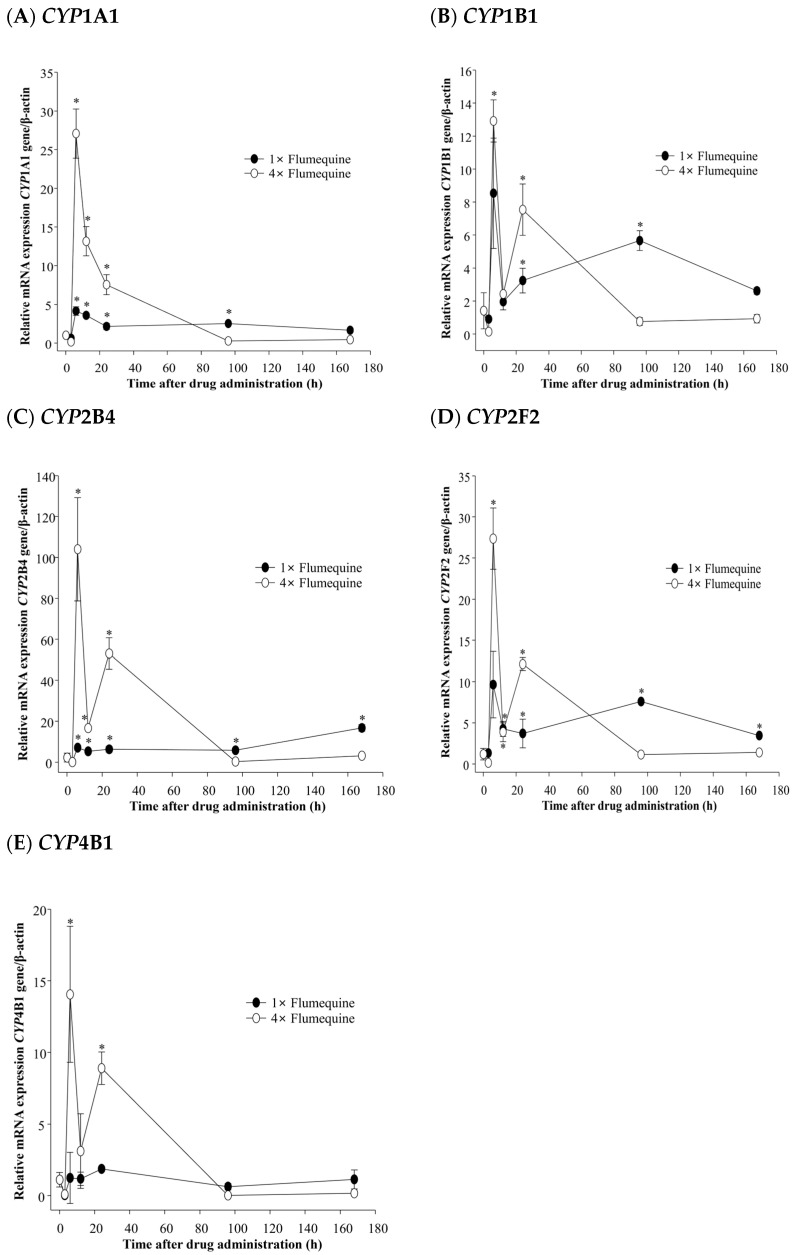
Gene expression of (**A**) *CYP*1A1, (**B**) CYP1B1, (**C**) CYP2B4, (**D**) *CYP*2F2, and (**E**) *CYP*4B1 in the liver at 3 h, 6 h, 12 h, 24 h, 96 h, and 168 h after administration of different concentrations of flumequine to olive flounder. * indicate significant differences (*p* < 0.05).

In the liver of olive flounder, gene expression of *CYP*1A1 was significantly induced after flumequine treatment. The 4× flumequine group exhibited higher expression levels than the 1× flumequine group at 6–24 h (*p* < 0.05). The highest *CYP*1A1 expression was observed at 6 h (27.1-fold) but remained significant between 12 h and 24 h (Figure 1A) (*p* < 0.05). The gene expression of *CYP*1B1 increased at 6 h following 1× and 4× flumequine administration compared to the control group. The expression level in the 4× group was 12.9-fold, which was higher than the 8.5-fold induction observed in the 1× treatment group (Figure 1B).

*CYP*2B4 expression was significantly increased in both treatment groups. In the 1× flumequine group, moderate increases were observed at all time points, ranging from 2.3 to 7.5-fold (*p* < 0.05). In contrast, the 4× group showed a 46.6-fold increase at 6 h, with sustained upregulation of 7.4 and 23.8-fold at 12 and 24 h, respectively (Figure 1C) (*p* < 0.05). In the case of *CYP*2F2, 1× flumequine significantly induced gene expression by 6.4-fold compared with that in the control group at 96 h (Figure 1D) (*p* < 0.05). Furthermore, 4× flumequine resulted in a 23.0-fold significant increase at 6 h, which was 3.6-fold higher than the peak expression observed with 1× (Figure 1D) (*p* < 0.05). No significant increase in *CYP*4B1 gene expression was observed in the 1× flumequine compared to that in the control. However, 4× flumequine significantly upregulated *CYP*4B1 expression by 12.7-fold and 8.1-fold at 6 h and 24 h after administration, respectively (Figure 1E) (*p* < 0.05).

### 3.2. Histopathological Analysis

**Figure 2:**
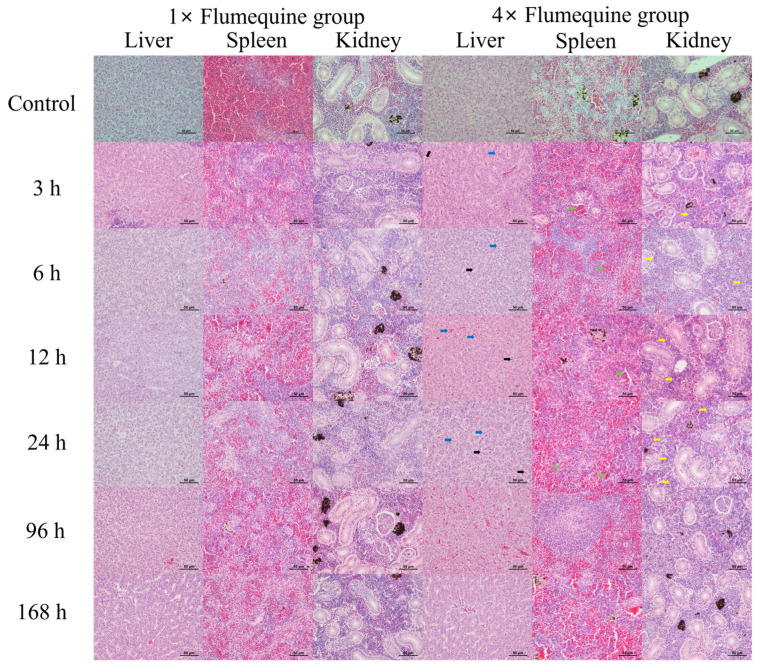
Histopathological sections of liver, spleen, and kidney after flumequine administration at 3 h, 6 h, 12 h, 24 h, 96 h, and 168 h in olive flounder. Sections were stained with hematoxylin and eosin to observe histopathological changes. The microphotographs of histopathological lesion were observed using a microscope (ECLIPSE Ni-U, Nikon, Tokyo, Japan; Scale bar = 50 μm).

In the control group, no histopathological changes were observed in the liver, spleen, and kidney. However, mild hepatic atrophy, lymphocytic infiltration in the spleen, and hematopoiesis in the kidney were observed at 3 to 168 h in the test group exposed to 1× flumequine (Figure 2). In the 4× test group, hepatic atrophy (black arrows) and congestion (blue arrows) were observed from 3 to 24 h, along with lymphocytic infiltration (green arrows) in the spleen and hematopoiesis (yellow arrows) in the kidneys (Figure 2). All lesions observed in the 4× flumequine group exhibited a progressive increase in severity over time.

## 4. Discussion

Flumequine is an antibiotic used to treat bacterial infections. In this study, we investigated the effect of flumequine on olive flounder by analyzing the expression of cytochrome P450 (*CYP*) family genes in the liver and conducting histopathological analyses of the liver, spleen, and kidneys after oral administration of different concentrations of flumequine. Exposure to chemical substances can influence the expression of *CYP*s in fish [14]. Fish may experience adverse effects on their organs because of exposure to chemicals, including pharmaceuticals [15]. In this study, flumequine, an antibacterial agent used to treat bacterial diseases, tended to increase the expression of *CYP*1A1, *CYP*1B1, *CYP*2B4, *CYP*2F2, and *CYP*4B1 following administration at a 4× dose compared to a 1× dose. Therefore, it has been shown that acute exposure of olive flounder to high concentrations of flumequine induces an increase in gene expression compared with lower concentrations.

In zebrafish, exposure to 2,3,7,8-tetrachlorodibenzo-p-dioxin (TCDD), which is used in chemical risk assessments for fish, has been reported to induce *CYP*1A and *CYP*1B gene expression in zebrafish embryos [16]. These reports anticipated a similar effect on *CYP*1 family genes in olive flounder following exposure to xenobiotics. In this study, following exposure to flumequine, the expression of *CYP*1A1 and *CYP*1B1 increased in the liver of olive flounder (Figure 1A,B). This also suggests that after the drug diffuses into the hepatocytes, it may bind to intracellular carrier proteins and enter the nucleus to stimulate mRNA transcription [17]. Exposure to the toxic compound 3,3′,4,4′,5-pentachlorobiphenyl (PCB126) affected the expression of *CYP*2 family genes in zebrafish, which are regulated by the xenobiotic-metabolizing receptor Pregnane X Receptor (PXR) [18]. Therefore, the observed increase in *CYP*2B4 and *CYP*2F2 expression following flumequine exposure may be attributed to PXR-mediated regulatory effects (Figure 1C,D). The *CYP*4 family is primarily expressed in the liver and intestine of the rare minnow (*Gobiocypris rarus*) [19]. *CYP*4B1 is involved in the metabolism of toxic xenobiotics, including valproic acid [20]. In this study, the observed increase in liver *CYP*4B1 expression suggests that flumequine may induce toxicity in olive flounder (Figure 1E).

Fish generally show increased expression of *CYP* family genes after acute exposure to chemical substances. However, chronic exposure leads to a decrease in the expression of the *CYP* gene family. This has been reported as an adaptation to contaminated environments [21]. In this study, *CYP* gene expression increased after the acute exposure of olive flounder to flumequine. In humans, the increase in *CYP* gene expression is the cause of liver injury induced by drug toxicity because the liver plays a central role in the metabolism of most drug [22]. Although the fish liver does not perfectly mirror the human liver, it exhibits physiological processes and drug metabolism pathways that are similar to those in humans. This makes it a valuable experimental model for studying drug-induced liver injuries [23]. Therefore, the increase in *CYP* expression in the liver of olive flounder exposed to flumequine is likely a response induced by drug detoxification [24].

Histopathological analysis is used to assess the effect of antibiotics on aquatic organisms and is an experimental technique used to identify tissue lesions from antibiotic exposure [25]. Oral administration of flumequine induces hepatic tumors in mice [26]. Fluoroquinolones are similar to flumequine. In a mouse experiment, orally administered fluoroquinolone caused follicular hyperplasia in the spleen and induced lymphocytic inflammation in the kidneys [27]. In the present study, olive flounder exposed to a low concentration (1×) of flumequine exhibited mild lesions in the liver, spleen, and kidneys. In contrast, the administration of a high concentration (4×) of flumequine resulted in the exacerbation of tissue lesions. These results suggest that the liver, spleen, and kidneys of olive flounder exhibited a more pronounced response to high concentrations of flumequine than to low concentrations. The differences in histopathological findings between mice and olive flounder following exposure to quinolone antibiotics may be attributed to variations in administered drug concentrations and fundamental species-specific differences between mammals and fish.

In this study, olive flounder exposed to 4× flumequine exhibited increased *CYP* gene expression and severe histopathological lesions compared to those exposed to 1× flumequine. The observed upregulation of *CYP* gene expression and histopathological tissue lesions in the liver are presumed to be caused by the widespread diffusion of drug toxins throughout the liver via the portal vein [8]. This suggests that the liver plays a critical role in the response to antibiotic exposure. Therefore, the expression of drug-metabolizing genes and tissue damage in olive flounder are not independent but rather involve interacting mechanisms. Further research is necessary to clarify the associations between gene expression and tissue lesion changes.

## 5. Conclusions

In this study, the expression of *CYP* drug-metabolizing genes increased following 4× flumequine exposure compared to 1× exposure. Furthermore, 4× flumequine exposure exacerbated the tissue lesion severity relative to 1× exposure. Therefore, a 4× concentration of flumequine can exacerbate adverse health effects in olive flounder compared to a 1× concentration. To ensure safe and healthy aquaculture organisms, using high concentrations of flumequine should be discouraged, and adhering to established dosage guidelines is essential. These results contribute to elucidating the effects of flumequine exposure on drug metabolism and general toxicity in olive flounder.

## Figures and Tables

**Table 1:** Primers for RT-qPCR used in this study.

Genes	Primer	Sequences (5′-3′)
β-actin	forward	GAGATGAAGCCCAGAGCAAGAG
	reverse	CAGCTGTGGTGGTGAAGGAGTAG
*CYP*1A1	forward	GATGAGGAGCTGTGGAAAGA
	reverse	AGACTTCATTTCGAGCGATG
*CYP*1B1	forward	ATGCAGCTGTTCCTTTTCAC
	reverse	TTTGACCTCCTCTGCACTTC
*CYP*2B4	forward	CACACATACAAGAGCGTTGC
	reverse	CCCATGAGCTCTGTGTCTTT
*CYP*2F2	forward	AAGCCTTCATGCCTTTCTCT
	reverse	GGTTTAGGGGTCTGAGTCGT
*CYP*4B1	forward	TGCCTGAAGGTTCTCTTGTC
	reverse	GTCTGACCGATGCAGTTTCT

## Data Availability

The data presented in this study are available upon request from the corresponding author.
